# Identification of RPL5 and RPL10 as novel diagnostic biomarkers of Atypical teratoid/rhabdoid tumors

**DOI:** 10.1186/s12935-018-0681-1

**Published:** 2018-11-20

**Authors:** Yanming Ren, Chuanyuan Tao, Xiliang Wang, Yan Ju

**Affiliations:** 1 Department of Neurosurgery, West China Hospital of Sichuan University, No. 37 Guo Xue Xiang, Chengdu, Sichuan China; 2 Beijing Institute of Genomics, Chinese Academy of Sciences, Beijing, China

**Keywords:** Atypical teratoid/rhabdoid tumors, Kidney rhabdoid tumors diagnosis, Bioinformatics, Dysregulation, Biomarker

## Abstract

**Background:**

Rhabdoid tumors (RTs) are aggressive tumors that occur most frequently in children under 2 years old, which often invade kidney (KRTs) and Center Nervous System, named Atypical teratoid/rhabdoid tumors (AT/RTs). RTs often progress fast and lead to a high lethality. RTs have a low incidence, we can hardly accumulate enough samples to elicit the diagnosis. More importantly, histologically, RTs present a host of neural, epithelial, mesenchymal, or ependymal patterns, which makes them rather variable and difficult to diagnose. Molecularly, RTs are diagnosed mainly on the lack of SMARCB1/INI1 protein expression, which, on the one hand, accounts for 75% of RTs, on the other hand, loss of expression of SMARCB1 is not exclusive to RTs. So, there is a need to find more accurate diagnose markers of RTs.

**Methods:**

In this study, we analyzed 109 samples including AT/RT, KRT and corresponding normal samples downloaded form NCBI GEO database. First, we identified the differentially expressed lncRNAs and PCGs in AT/RT, KRT and corresponding normal samples. Second, we evaluated the co-expression relationship between lncRNA and PCG, and defined four types of the dysregulated PCG-lncRNA pairs. Third, we compared the differentially expressed genes, the dysregulated PCG-lncRNA pairs and commonly known cancer genes, we get potential diagnostic markers. Then, the potential diagnostic markers were subjected to Receiver operating characteristic (ROC) analysis to assess the diagnostic accuracy. Importantly, differential expression of the marker genes in different tumors was shown to distinguish AT/RT and KRT from other pediatric tumors specifically.

**Results:**

We compared the expression profiles between 47 AT/RTs, 31 KRTs, 8 normal brain samples, and 23 normal kidney samples. After applying a stringent set of criteria on the gene expression profiles, we identified 3667 PCGs and 81 lncRNAs differentially expressed in AT/RT, 3809 PCGs and 34 lncRNAs differentially expressed in KRT tissues. Next, we compared the three sets(AT/RT versus control brain samples, KRT versus control kidney samples, and AT/RT versus KRT) of differentially expressed lncRNAs and PCGs, 491 PCGs and 2 lncRNAs appeared in all three sets. We examined the correlation of the expression levels of these genes in the ‘three-set overlap’ group and identified four types of dysregulated lncRNAs and PCGs. By compared these genes to the well-known cancer driver genes, 19 PCGs were selected as potential candidates of diagnostic markers. Filtered with the number of the corresponding co-expressed lncRNA (namely “degree”), eight PCGs with more than five lncRNAs in the ‘three-set overlap’ group were selected as candidate diagnostic markers. Among them, RPL5 and RPL10 exhibited high sensitivity and specificity in diagnosis of AT/RT and KRT. However, when these two genes were used to distinguish AT/RT and KRT from other pediatric tumors, only AT/RT can be distinguished from medulloblastoma.

**Conclusions:**

Our study mined existing GEO datasets for novel diagnostic markers associated with Rhabdoid tumors, and identified RPL5 and RPL10 as potential diagnostic markers for AT/RT. These two biomarkers may be used as supplementary biomarkers to canonical diagnostic tools such as biopsy and immunohistochemistry.

**Electronic supplementary material:**

The online version of this article (10.1186/s12935-018-0681-1) contains supplementary material, which is available to authorized users.

## Background

Rhabdoid tumors (RTs) are aggressive tumors that occur most frequently in children under 2 years old. RTs often occur in the kidney (KRTs) or the central nervous system (CNS), which are termed Atypical teratoid/rhabdoid tumors (AT/RTs). Extracranial RTs were first recognized as a physiological entity nearly 40 years ago [1]. Later, Haas and colleagues introduced the term rhabdoid in describing KRT, due to the close histological resemblance of the tumor cells to rhabdomyoblasts, although subsequent studies have not confirmed a myogenic origin of these tumor cells [2]. In 1987, AT/RT was recognized as a discrete clinical entity based on pathologic and genetic characteristics [3]. Prior to that, it had been mostly classified as either medulloblastoma, primitive neuroectodermal tumor, or choroid plexus carcinoma. Following this description, the World Health Organization (WHO) began to classify AT/RT as an embryonal grade IV neoplasm in 1993 [4].

Epidemiologic studies of RT have been limited by the fact that this is a rare disease. So far there have been only a handful of epidemiologic reports. In a study conducted in the UK, 106 children under 15 years old were diagnosed with extracranial RT in the UK between in a period of nearly 20 years [5], resulting in an age-standardized annual incidence of 0.6 per 1 million children. In the US, several studies observed that AT/RT accounted for 1–2% in pediatric brain tumors, and for 4.4% of CNS tumors in children aged zero to 5 years [6–9]. Two more recent surveys conducted in China draw consistent results of a prevalence of AT/RT at approximately 5% in pediatric CNS tumors, which is comparable to that in the US study.

Aside from low incidence rate, there are other factors that poses challenges to the diagnosis and treatment of RTs. Histologically, RTs manifest several characteristic features, including eosinophilic cytoplasm, large nucleoli, and filamentous cytoplasmic inclusions. The tumors may present a host of neural, epithelial, mesenchymal, or ependymal patterns, which makes them rather variable and difficult to diagnose [10]. Moreover, RTs often progress fast and lead to a high lethality. In the UK study of extracranial RT, 1-year survival was 31% [5]. The patients usually suffers from metastasis and, to make things worse, the young age of patients limits use of radiotherapy. In an early report of 22 cases of KRTs in children, metastases were found in 82% of cases, either at diagnosis, or developing from 2 weeks to 9 months after diagnosis. Only two patients eventually survived, both with localized disease (stage II) [11]. Therefore, early diagnosis of this formidable disease is of key importance and in urgent demand.

Currently RTs are diagnosed mainly on immunohistochemistry (IHC) results, specifically, the lack of SMARCB1/INI1 protein expression, or less frequently, that of SMARCA4/BRG1 protein expression [4]. Initial genetic studies suggested that approximately 75% of RTs are characterized by biallelic inactivation of the *SMARCB1* locus, which indicated a sensitivity of close to 75% [12]. However, loss of expression of SMARCB1 is not exclusive to RTs, but also has been observed in other types of cancers, including chordoma, epithelioid sarcoma, cribriform neuroepithelial tumor, and medullary renal cell carcinoma [13–19]. Together, these lines of evidence suggest that SMARCB1 expression alone is neither sufficiently sensitive nor specific for diagnosing RTs. Moreover, in particular for CNS AT/RTs, another severe limitation in clinical diagnosis is the potential misdiagnosis as medulloblastomas (MBs) or primitive neuroectodermal tumors (PNETs), owing to the close histological resemblance of the rhabdoid cells and neuroepithelial tissue in these tumors [3, 20]. In conclusion, diagnostic markers with improved sensitivity and specificity are needed to complement the current practice, to the end of developing a comprehensive diagnostic strategy with enhanced sensitivity and precision.

In this study, we set out to identify diagnostic markers for RTs by employing a molecular profiling approach. Protein coding genes (PCGs) and long non-coding RNAs (lncRNAs) showing aberrant expression in AT/RT and KRT cases were identified, respectively, and the co-expression between these significantly dysregulated genes was evaluated. Through further comparison of differentially expressed genes, the dysregulated PCG-lncRNA pairs, and commonly known cancer genes, candidate diagnostic markers for AT/RT were identified and subjected to Receiver Operating Characteristic analysis to assess the performance of these candidates. Two PCGs, RPL5 and PRL10, exhibited high sensitivity and specificity not only in diagnosis of AT/RT but also differential diagnosis of AT/RT and KRT, as therefore show considerable promise for AT/RT diagnosis, and warrants further investigation.

## Methods

### Data analysis

The raw data were downloaded from the NCBI GEO database (GSE15641, GSE11482, GSE30946, GSE64019, GSE28026, GSE35493, GSE64019, GSE70421, GSE35493). The limma package was used to deal with the raw data in CEL format, with MAS5 algorithm, to quantify expression level and to identify the difference of gene expression. The biomaRt package was used to convert the probe ID to Ensembl ID. Genes were categorized as “protein coding” and “long non-coding” based on an Ensembl annotation file in the GTF format. Among non-coding genes, rRNAs, tRNAs, miRNAs, snoRNAs and other known classes of RNAs were excluded, and lncRNAs were defined as all non-coding genes longer than 200 nucleotides and not belonging to other RNA categories.

### Pearson’s correlation coefficient

Pearson’s correlation coefficient (PCC) was calculated by in-house R- scripts and was utilized to evaluate the co-expression relationship between lncRNA and PCG. Co-expressed pairs were defined with a cutoff of |PCC| ≥ 0.7 and P < 0.001.

### Data visualization

Unsupervised hierarchical clustering was done by R software (version 3.3.2, http://www.r-project.org/). The receiver operating characteristic (ROC) and the area under the ROC curves (AUC) values were obtained from the pROC package. Unless otherwise specified, data were analyzed and visualized using R software (version 3.3.2).

### Enrichment analysis

For enrichment analysis to explore their biological effects, PCGs were analyzed using the clusterProfiler package. The GO terms and KEGG pathways with p values or FDR of < 0.05 were considered as significantly enriched function annotations.

### Differential RPL5/10 expression analysis across Affymetrix datasets

We downloaded GSE85217 and GSE2712 from GEO dataset. GSE85217 contains 762 medulloblastoma patients expression data, and GSE2712 contains 18 Wilms’ tumors and 14 clear cell sarcoma of the kidney. The former used Affymetrix Human Gene 1.1 ST Array, the latter used Affymetrix Human Genome U133A Array. So in order to make the data comparable, we used the Array Generation based gene Centering (AGC) method to compare the expression value of RPL5/10 between different datasets [21]. The AGC method scaled datasets with a scaling factor that is defined based on the housekeeping genes.

## Results

### Transcriptome expression profiles in AT/RT, KRT and normal samples

**Fig. 1:**
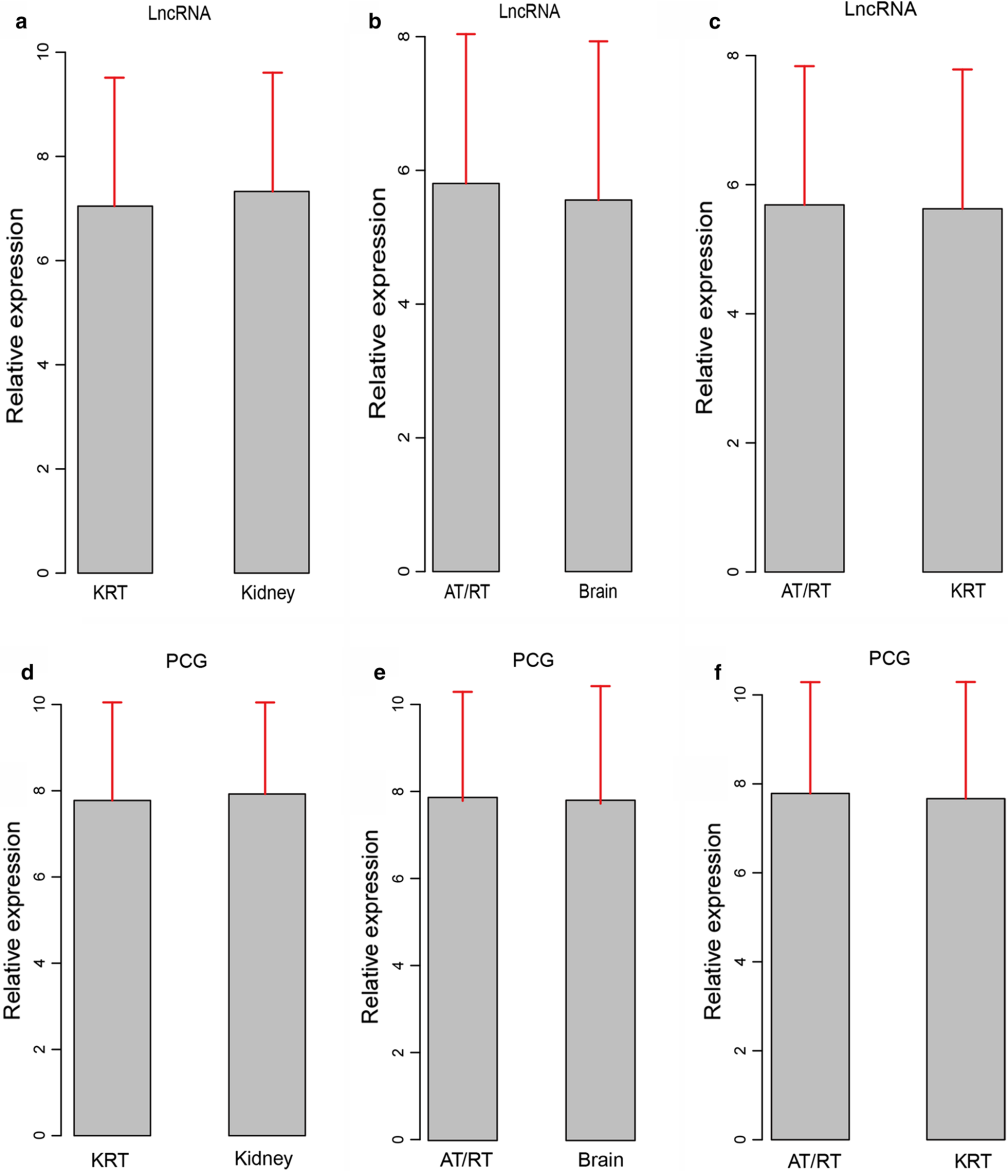
Expression profiles of lncRNAs and PCGs in tumor and normal samples. Relative expression fold changes of lncRNAs in kidney versus KRT (**a**), AT/RT versus Brain (**b**), and AT/RT versus KRT (**c**) are presented, along with relative PCG expression in kidney versus RT (**d**), AT/RT versus Brain (**e**), AT/RT versus KRT (**f**)

We started by comparing the expression profiles between 47 AT/RTs, 31 KRTs, 8 normal brain samples, and 23 normal kidney samples (sample list in Additional file 1: Table S1). Between tumor and normal samples, expression of lncRNAs showed greater level of alteration in AT/RT or KRT (Fig. 1a, b) than that of PCGs (Fig. 1d, e), suggesting a specific expression pattern of lncRNAs in these tumors. However, comparing with KRT, both lncRNAs and PCGs showed weaker changes in expression levels in AT/RT (Fig. 1c, f), suggesting that resemblance in the expression profiles of tumors of RTs and its subtype, AT/RT.

### Differentially expressed lncRNAs and PCGs in AT/RT, KRT and normal samples

After applying a stringent set of criteria on the gene expression profiles, we identified groups of lncRNAs and PCGs differential expressed between tumor and normal tissue samples (p = 0.00001 and fold change (FC) = 2 for KRT vs. kidney and AT/RT vs. brain, p = 0.001 and fold change = 2 for KRT vs. AT/RT). In total, we identified 3667 PCGs and 81 lncRNAs differentially expressed in AT/RT, with 988 up-regulated and 2679 down-regulated PCGs and 14 up-regulated and 67 down-regulated lncRNAs in the tumor samples (Table 1). Notably, there were more than twice as many down-regulated genes as up-regulated ones. Between KRT and normal samples, 3809 PCGs (1963 up-regulated and 1846 down-regulated) and 34 lncRNAs (14 and 20, respectively) showing aberrant expression in KRT tissues (Table 1). As differentially expressed genes between KRT and AT/RT, 3381 PCGs and 91 lncRNAs showed significantly altered expression levels. Among these genes we identified 2568 up-regulated and 813 down-regulated PCGs along with 59 up-regulated and 32 down-regulated lncRNAs (Table 1). Of note, there were approximately three times as many down-regulated genes as up-regulated ones, suggesting the significance of these genes in differentiating RTs and its subtype AT/RT.

**Table 1:** A summary of differentially expressed lncRNAs and PCGs between AT/RT, KRT and the corresponding normal control samples

	AT/RT vs. Brain		KRT vs. Kidney		KRT vs. AT/RT	
	Up	Down	Up	Down	Up	Down
PCG	988	2679	1963	1846	2568	813
lncRNA	14	67	14	20	59	32

*AT/RT* atypical teratoid/rhabdoid tumors, *KRT* kidney rhabdoid tumors)

**Fig. 2:**
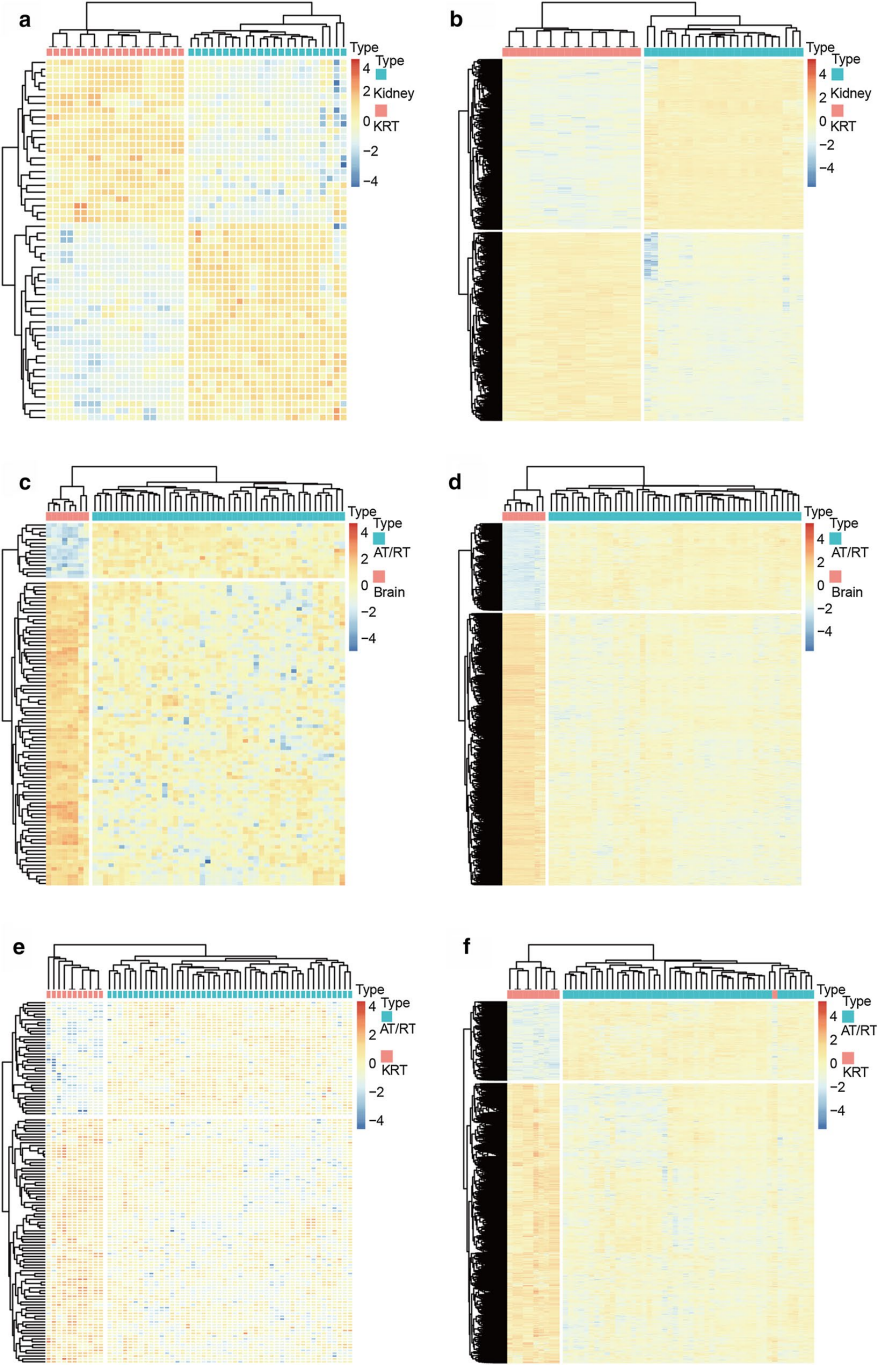
Hierarchical clusters of significantly dysregulated lncRNAs and PCGs revealed distinct expression patterns in KRT vs. Kidney (**a**, **b**), AT/RT vs. Brain (**c**, **d**), AT/RT vs. KRT (**e**, **f**)

A hierarchical cluster analysis of differentially expressed lncRNAs and PCGs showed that samples derived from AT/RT or KRT were well distinguished from corresponding normal ones based on the expression patterns of these genes (Fig. 2). This clear distinction between tumor and control samples suggests the highly specific nature of the dysregulation of these genes to the corresponding diseases.

**Fig. 3:**
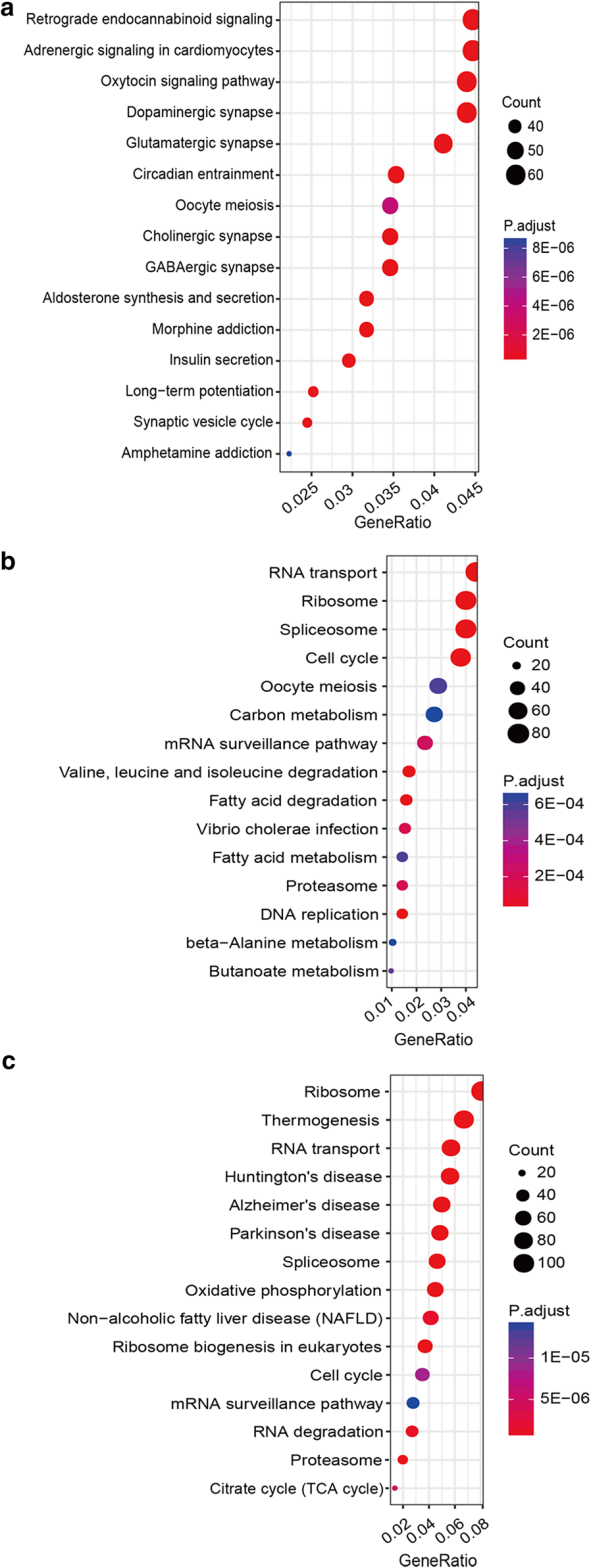
KEGG pathway enrichment analysis of PCGs that showed significantly differential expression in AT/RT vs. Brain (**a**), KRT vs. Kidney (**b**), and KRT vs. AT/RT (**c**)

Next, we performed pathway enrichment analysis on differentially expressed PCGs to gain insights into pathways potentially implicated in this disease. A total of 72 pathways were significantly enriched (adjusted p value < 0.05). As show in Fig. 3a, many of the differentially expressed PCGs in AR/RT play roles in neural signaling pathways, such as retrograde endocannabinoid signaling (endocannabinoids serve as retrograde messengers at synapses in various regions of the brain [22, 23]), dopaminergic synapse (Dopamine is an important and prototypical slow neurotransmitter in the mammalian brain, where it controls a variety of functions including locomotor activity, motivation and reward, learning and memory, and endocrine regulation [24, 25]), glutamatergic synapse (Glutamate is the major excitatory neurotransmitter in the mammalian central nervous system [26, 27]), “Cholinergic synapse” (Acetylcholine is a neurotransmitter widely distributed in the central nervous system [28, 29]), GABAergic synapse (Gamma aminobutyric acid (GABA) is the most abundant inhibitory neurotransmitter in the mammalian central nervous system [30, 31]). Differentially expression PCGs in KRT, on the other hand, were enriched mainly in processes related to RNA transcription and protein translation, such as RNA transport, ribosome, and spliceosome (Fig. 3b). As for the differentially expressed PCGs between AT/RT and KRT, 19 pathways were enriched significantly (adjusted p value < 0.05). Among these enriched pathways, some overlapped with ones enriched from dysregulated genes in KRT (Fig. 3b). In addition, there were also a considerable number of pathways involved in neurodegenerative diseases, such as Huntington’s disease, Alzheimer’s disease, and Parkinson’s disease (Fig. 3c). As AT/RT and KRT are both subtypes of RT, the overlapping enriched pathways may represent common pathological mechanisms in both subtypes, while the more CNS-specific pathways may be specific to AT/RT.

**Fig. 4:**
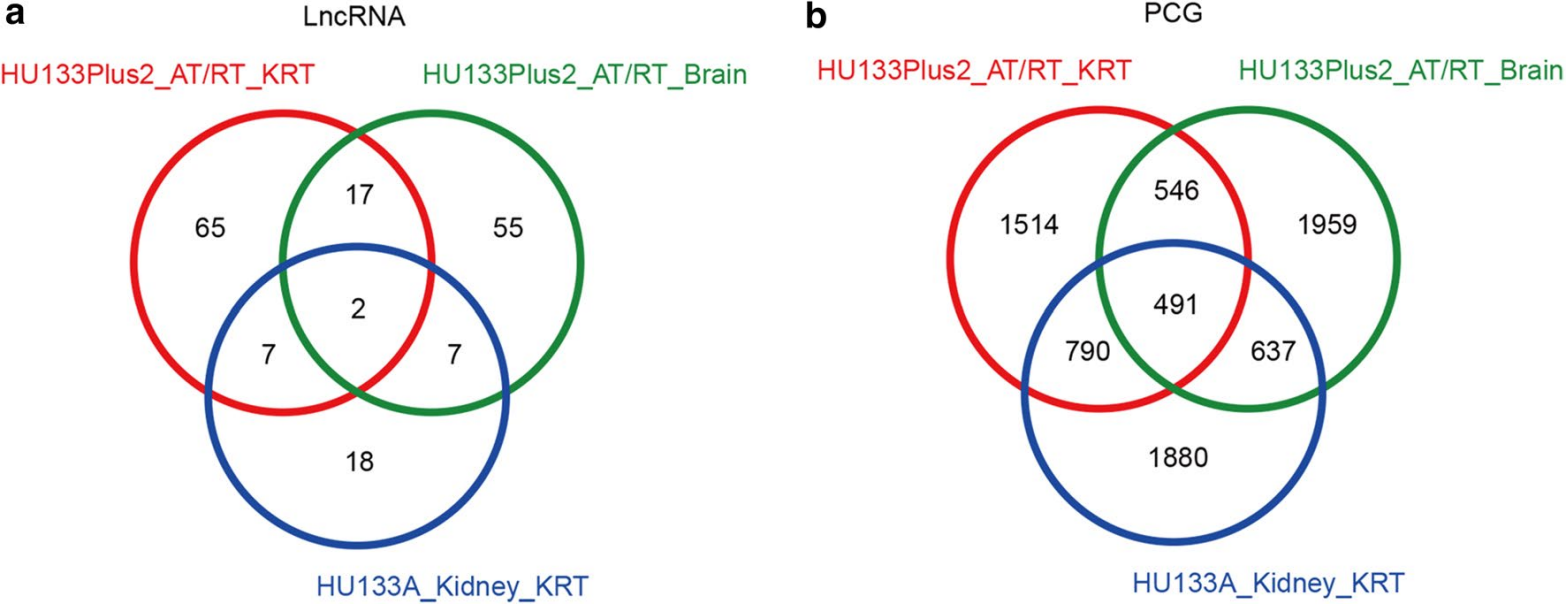
Differentially expressed lncRNAs (**a**) and PCGs (**b**) significantly dysregulated in AT/RT vs. Brain, KRT vs. Kidney and KRT vs. AT/RT

Next, we compared the three sets of differentially expressed lncRNAs and PCGs, namely those showing significantly different expression levels between AT/RT versus control brain samples, KRT versus control kidney samples, and AT/RT versus KRT. Venn diagrams were plotted for differentially expressed lncRNAs (Fig. 4a) and PCGs (Fig. 4b), and genes that appeared in all three sets (referred to as the ‘three-set overlap’ group) were selected. A total of 491 PCGs and 2 lncRNAs fell in this group, which served as the pool for further screening of candidate markers for diagnosing AT/RT.

### Dysregulated network of differentially expressed features

Following the identification of differentially expressed lncRNAs and PCGs in AT/RT and KRT, we examined the correlation of the expression levels of these genes in the ‘three-set overlap’ group. A Pearson’s correlation coefficient (PCC) was calculated for the expression levels of each pair of lncRNAs and PCGs across disease states. There were a total of 12,831 PCGs in the microarray profiles (denoted AllPCG in Table 2), among which 491 differentially expressed PCGs (denoted DiffPCG) (Fig. 4b) in all ‘three-set overlap’ group. Specifically, among these DiffPCGs we focused on the genes reported to be strongly associated with cancer (denoted CancerG). (“cancer genes” were cited from the report of Science [32]; “CancerG” for short, Additional file 2: Figure S1).

**Table 2:** Four types of dysregulated pairs of lncRNA-PCG in AT/RT vs. Brain, KRT vs. Kidney and AT/RT vs. KRT

AT/RT vs. Brain					
Brain	AT/RT	Type	AllPCG	DiffPCG	CancerG
Yes	No	I	66,125	18,651	2629
No	Yes	II	3059	232	138
Positive	Nagetive	III	16	4	2
Nagetive	Positive	IV	36	2	4
KRT vs. Kidney					
Kidney	KRT	Type	AllPCG	DiffPCG	CancerG
Yes	No	I	16,408	6259	675
No	Yes	II	28,350	7371	1284
Positive	Nagetive	III	260	100	12
Nagetive	Positive	IV	157	35	5
AT/RT vs. KRT					
AT/RT	KRT	Type	AllPCG	DiffPCG	CancerG
Yes	No	I	5543	776	263
No	Yes	II	45,245	6908	1742
Positive	Nagetive	III	53	2	5
Nagetive	Positive	IV	21	3	1

Co-expressed pair were classified into four types, based on presence and type of regulation of the co-expression in the three sets of comparisons, namely (A) AT/RT vs normal brain samples, (B) KRT vs normal kidney samples, and (C) AT/RT vs KRT samples. Type I: co-expressed pairs that were present in AT/RT (A), KRT (B), and AT/RT (C), and absent in normal brain samples (A), normal kidney samples (B), and KRT(C). Type II: co-expressed pairs that were absent in AT/RT (A), KRT (B), and AT/RT (C), and was present in the corresponding control samples. Type III: co-expression pairs that were positively co-expressed in AT/RT (A), KRT (B), and AT/RT (C) and negatively co-expressed in the corresponding control samples. Type IV: co-expression pairs that were negatively co-expressed in AT/RT (A), KRT (B), and AT/RT (C) and positively co-expressed in the corresponding control samples

We identified four types of dysregulated lncRNAs and PCGs in Table 2. As listed, there were 69236, 18889, and 2773 dysregulated pairs of “AllPCG”, “DiffPCG”, and “CancerG” in AT/RT, respectively. The overwhelming majority type of the dysregulated pairs was Type I (Table 2) illustrating a massive loss in regulation of lncRNAs to PCGs in AT/RT patients. There were 45175, 13,765, and 1976 dysregulated pairs of “AllPCG”, “DiffPCG”, and “CancerG” in KRT vs. Kidney, respectively. It was the opposite that the overwhelming majority type of the dysregulated pairs was Type II in “AllPCG”, “DiffPCG”, and “CancerG”, especially in “CancerG” (Table 2). In AT/RT vs. KRT There were 50,862, 7689, and 2011 dysregulated pairs of “AllPCG”, “DiffPCG”, and “CancerG”, respectively, with the majority grouped into Type II (Table 2), showing vast difference of lncRNA dysregulation in AT/RT and KRT. The four types of dysregulated pairs may be one important reason for the aberrance of cancer cells, they may also play important roles in AT/RT or KRT, as well.

**Fig. 5:**
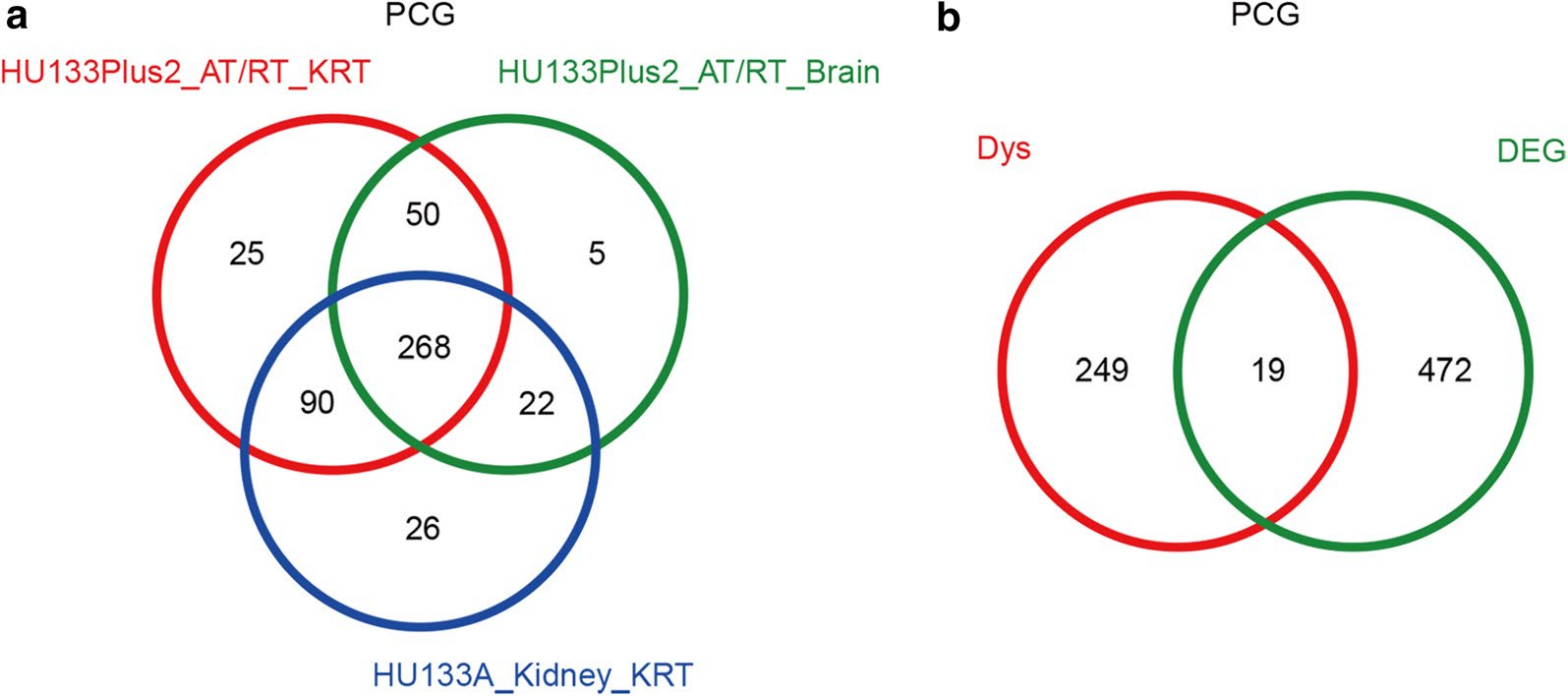
Selection of differentially expressed cancer-related PCGs. (**a**) dysregulated CancerG between AT/RTvs. Brain, KRT vs. Kidney, and AT/RT vs. RT. **a** A total of 268 CancerG were identified; **b** Intersect the 268 CancerG with 491 DiffPCG, 19 genes were identified belong to both CancerG and DiffPCG

**Fig. 6:**
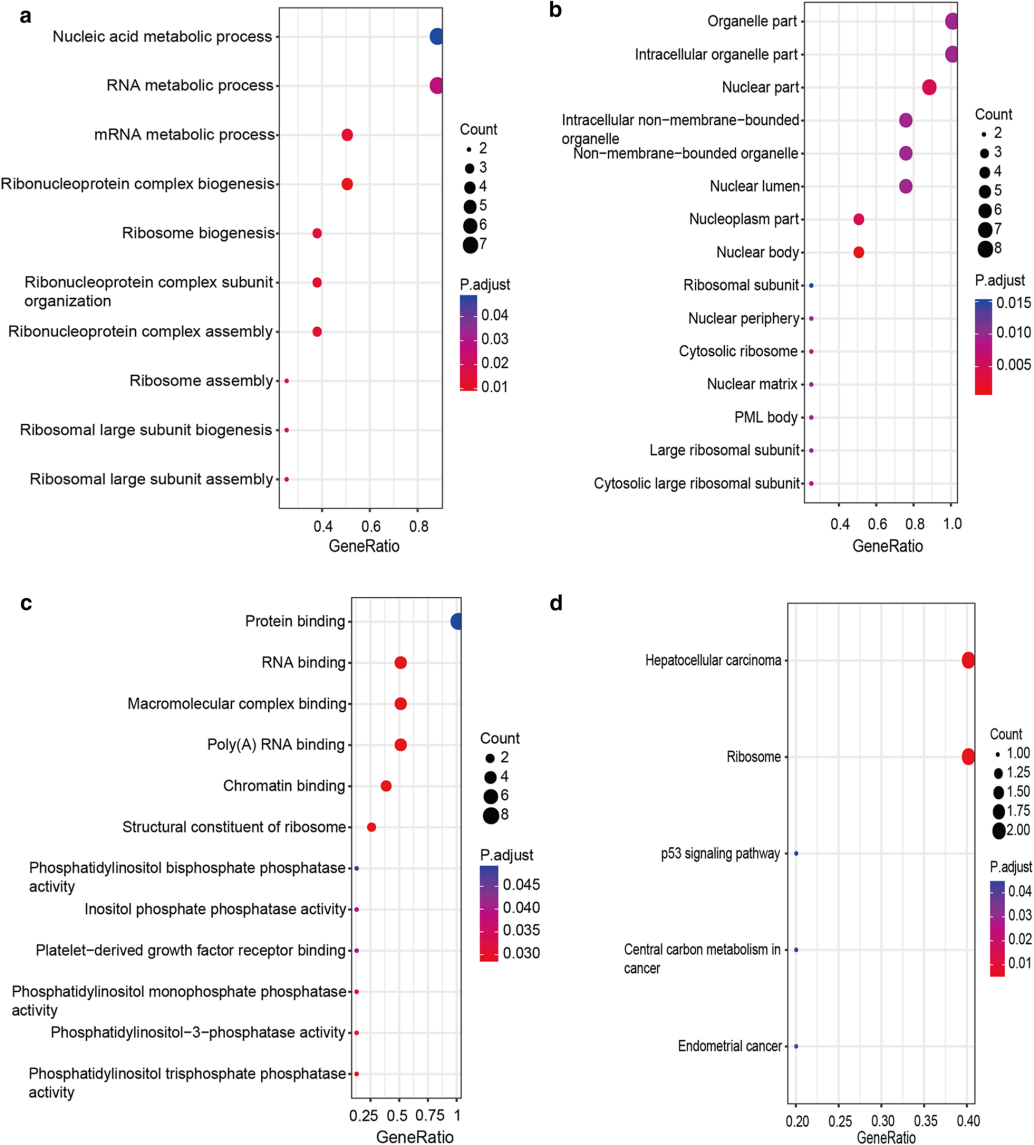
KEGG pathway and GO enrichment of the eight differentially expressed cancer-related PCGs. **a** Enriched GO terms related to biological process, **b** enriched GO terms related to cellular components, **c** enriched GO terms related to molecular functions, and **d** enriched KEGG pathways

We focused on the “cancer genes” because they have been established to show high relevance in cancer initiation and development. We compared the dysregulated cancer genes in AT/RT vs. Brain, KRT vs. Kidney and AT/RT vs. KRT, there were 268 “cancer genes” dysregulated in all of the ‘three-set overlap’ group (Fig. 5a). Next, we checked whether those 268 genes were also differentially expressed in the ‘three-set overlap’ group. After comparing with the 491 DiffPCG (Fig. 4b), 19 PCGs were selected as potential candidates of diagnostic markers (Fig. 5b and Table 3). The dysregulated co-expression pairs were retrieved for these 19 PCGs, and the number of the corresponding co-expressed lncRNA (namely “degree”) was showed in Table 4, where a high number is indicative of the complexity of lncRNA regulation to which the corresponding PCG is subjected, and suggests a more central position in the co-expression network. Therefore, eight PCGs with more than five lncRNAs in the ‘three-set overlap’ group were selected as candidate diagnostic markers, including RPL5, RPL10, NONO, PBRM1, PCM1, PTEN, SF3B1, and ZMYM2. KEGG pathway and Gene Ontology enrichment analysis highlighted ribosome as the main convergence of these aberrantly expressed genes, strongly hinting at a significant role of dysregulation of ribosome-related functions and processes in AT/RT and KRT (Fig. 6).

**Table 3:** List of 19 cancer-related PCGs that showed significantly altered expression in AT/RT vs. Brain, KRT vs. Kidney and KRT vs. AT/RT

Symbol	Entrezgene
ATIC	471
DDB2	1643
FANCA	2175
GNAS	2778
NONO	4841
NPM1	4869
PBRM1	55,193
PCM1	5108
PDE4DIP	653,513
PTEN	5728
RPL10	6134
RPL22	6146
RPL5	6125
SF3B1	23,451
SMARCE1	6605
SRSF3	6428
SUZ12	23,512
TCF3	6929
ZMYM2	7750

**Table 4:** The dysregulated co-expression pairs retrieved for the 19 PCGs, and the number of the corresponding co-expressed lncRNA (namely “degree”)

AT/RT vs. KRT	Degree	AT/RT vs. Brain	Degree	KRT vs. Kidney	Degree
ATIC	5	ATIC	6	GNAS	7
FANCA	7	GNAS	13	NONO	7
NONO	7	NONO	19	NPM1	10
NPM1	11	PBRM1	21	PBRM1	8
PBRM1	6	PCM1	6	PCM1	13
PCM1	8	PDE4DIP	11	PTEN	11
PDE4DIP	9	PTEN	15	RPL10	11
PTEN	13	RPL10	9	RPL22	12
RPL10	7	RPL5	6	RPL5	14
RPL5	8	SF3B1	26	SF3B1	12
SF3B1	13	SUZ12	21	SRSF3	5
SMARCE1	5	ZMYM2	26	TCF3	7
SRSF3	6	RPL22	4	ZMYM2	12
SUZ12	5	NPM1	3	DDB2	3
TCF3	8	DDB2	2	PDE4DIP	3
ZMYM2	13	SRSF3	2	SMARCE1	3
RPL22	4	TCF3	2	SUZ12	2
GNAS	2	FANCA	1	ATIC	1
DDB2	1	SMARCE1	1	FANCA	1

### Diagnostic values of RPL5 and RPL10 in AT/RT and KRT

To evaluate the performance of the eight candidate markers identified in the last section in diagnosing AT/RT and RT, ROC (Receiver operating characteristic) analysis was performed and the area under curve (AUC) served as the basis for selecting the most sensitive and specific candidates.

**Fig. 7:**
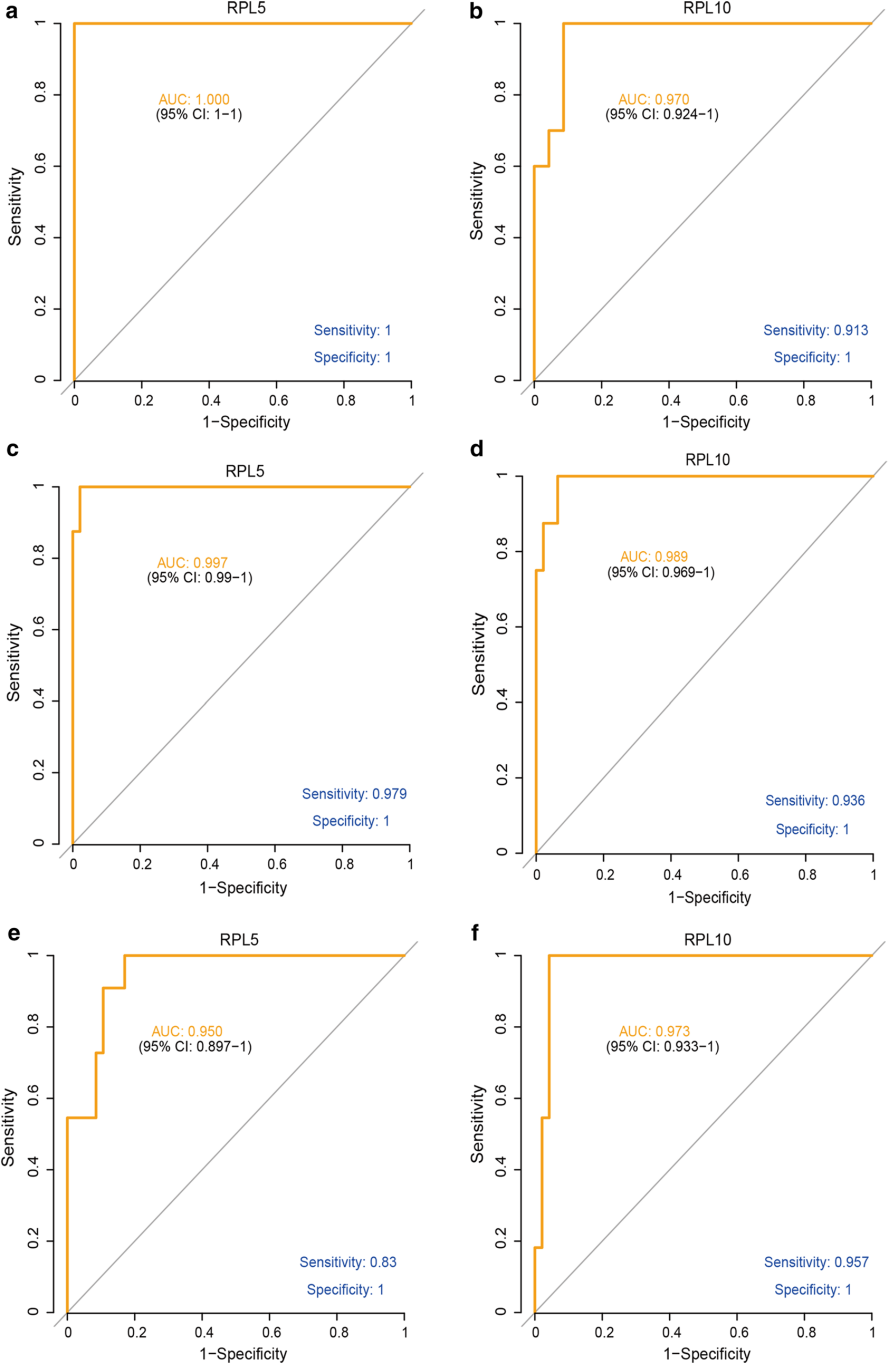
ROC analysis of assessing the performance of RPL5 and RPL10 as diagnostic markers of AT/RT and KRT. The left column shows ROC analysis of RPL5 in diagnosing **a** KRT, **c** AT/RT, and **e** distinguishing AT/RT from KRT. The right column shows ROC analysis of RPL10 in diagnosing **b** KRT, **d** AT/RT, and **f** distinguishing AT/RT from KRT

Two significantly deregulated cancer-related genes, RPL5 and RPL10, showed outstanding performance in the ROC analysis. As shown in Fig. 7, in KRT versus normal kidney samples, AUC for RPL5 reached 1 (95% CI 1–1), with both sensitivity and specificity level at 1 (Fig. 7a). Also, the AUC of RPL10 reached 0.97 (95% CI 0.924–1), with a sensitivity level of 0.913 and specificity level of 1, respectively (Fig. 7b). Moreover, high levels of diagnostic values were also observed for both genes in AT/RT. AUCs were 0.997 (95% CI 0.99–1) and 0.989 (95% CI 0.969–1) for RPL5 and RPL10, respectively, with sensitivity levels of 0.979 (RPL5) and 0.936 (RPL10), and a specificity level of 1 for both genes (Fig. 7c, d).

More importantly, both genes were powerful indicators for distinguishing AT/RT from KRT. When comparing expression profiles in AT/RT samples with those in KRT samples, AUC for RPL5 was 0.950 (95% CI 0.897–1) with sensitivity and specificity levels of 0.83 and 1, respectively (Fig. 7e). AUC for RPL10 was 0.973 (95% CI 0.933–1) and also with high sensitivity (0.957) and specificity (1) (Fig. 7f). In other words, in the samples examined in this analysis, AT/RT and KRT could be accurately diagnosed based on expression levels of RPL5 and RPL10 (Additional file 3: Figure S2).

### RPL5 and RPL10 can be used to distinguish AT/RT from medulloblastoma

**Fig. 8:**
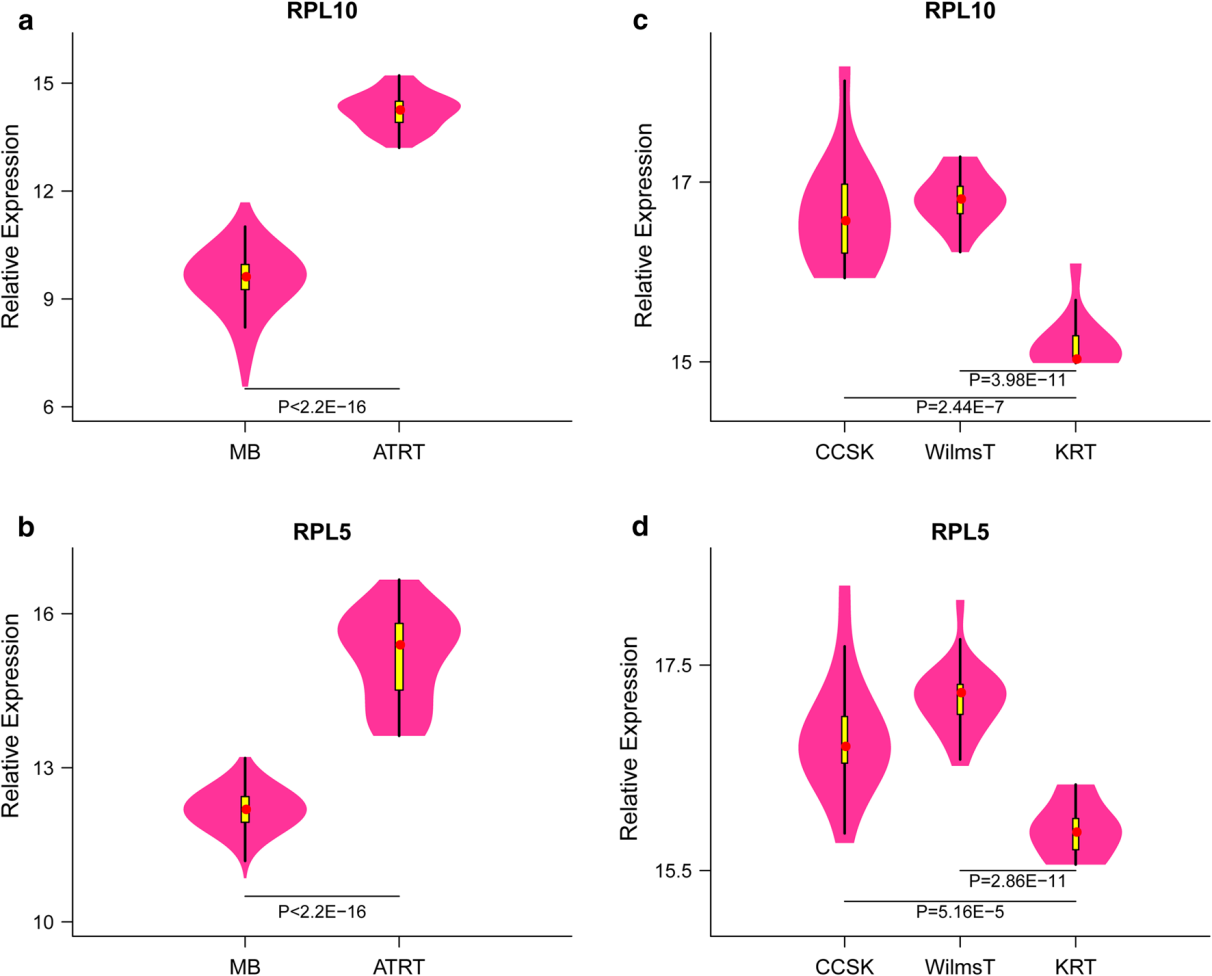
RPL5/10 expression between AT/RT and medulloblastoma (**a**, **b**), KRT, Wilm’s tumor and clear cell sarcoma of the kidney (**c**, **d**)

To evaluate whether RPL5 and RPL10 can be used to distinguish AT/RT and KRT from other tumors, we compared the expression levels of RPL5 and RPL10 in RTs and other types of tumor. Compared with medulloblastoma, RPL5 and RPL10 were signifcantly upregulated in AT/RT, with fold changes of 1.25 and 1.5 (p < 0.001, Fig. 8a, b), respectively. On the other hand, when compared with Wilms’ tumor and clear cell sarcoma of the kidney, RPL5 and RPL10 were signifcantly downregulated in KRT (Fig. 8c, d). Together, these results suggest that RPL5 and RPL10 as promising diagnostic markers not only in distinguishing for AT/RT from normal tissues but also in from other types of pediatric tumors.

## Discussion

Rhabdoid tumors are highly lethal cancers that most frequently observed in young children. Research into the diagnosis and treatment has been hampered by the rare nature of this disease despite its urgency. In a recent study, Chun et al. performed a molecular dissection of Malignant rhabdoid tumors (MRT, mainly KRT) using RNA sequencing [33]. Expression profiles of 40 primary extra-cranial malignant rhabdoid tumors, three human embryonic stem cell lines, and four fetal cerebellum samples were collected and screened for aberrantly expressed genes. Through compare RTs gene expression with genes expressed both in cell lines and fetal cerebellum samples, Author identified 398 up-regulated genes and 615 down-regulated ones. These genes may be used as diagnosis markers of MRT, but this study did not focus on identifying marker candidates for KRT diagnosis. More specifically, similar investigations were conducted in AT/RT over the past few years. Based on patterns in the transcriptional profile, Torchia and colleagues [34] classified AT/RT into three subgroups with distinct genomic profiles, implicated cellular processes, and clinicopathological and survival features. These findings were consistent with those of an independent study [35]. All three reports, however, focused on the classification and prognosis and AT/RT. Chakravadhanula et al. [36] evaluated the performance of HOTAIR and HOXC as diagnostic markers of AT/RT, however, the authors found that both genes are not sufficient for distinguishing AT/RT from several other forms pediatric brain tumors. In an interesting study, Ho et al. [37] proposed three oncogenes, FGFR2, S100A4 and ERBB2 (HER2/neu), as markers for diagnosing AT/RT, based on the aberrant high expression in tissue samples expressing SMARCB1. Overexpression of these genes may be used as novel markers that complement the current criteria, lack of SMARCB1expression. However, as these results were derived from a limited number of samples, further research is warranted to validate these candidates.

Regulation of PCG expression have been known to occur through a number of mechanisms. Upstream regulators include microRNAs and lncRNAs. In an interesting study into the role of microRNAs in Grupenmacher et al. [38] analyzed the expression profiles of microRNA and PCGs in 13 AT/RT and 10 KRT cases, as well as two human RT cell lines. They found 122 genes significantly differentially expressed between AT/RT and KRT, about 76.22% (93/122) of which down regulated in AT/RT, which was in accordance with our result (Table 1). However, the authors reported a general lack significantly altered expressions in microRNAs between AT/RT and KRT, Therefore, we focused on elucidating the potential of significantly altered lncRNA expression in our investigation, rather than miRNA, as lncRNAs have recently been established as key regulators in cancer. Through identifying differentially expression lncRNAs and constructing lncRNA-PCG co-expression network, 19 PCGs were selected based on co-expression relationship. Further screening, based on numbers of co-expressing lncRNAs, provided a final list of eight candidate markers.

Both *RPL5* and *RPL10* encode members of the 60S subunit of the ribosome [39, 40]. The protein expression of both genes is relative low in the normal brain [41]. RPL5 binds 5S rRNA and forms a stable complex, the 5S ribonucleo protein particle, which is necessary for the 5S rRNA transport, where cytoplasmic 5S rRNA is transported to the nucleolus to be assembled into ribosomes. RPL5 may inhibit tumorigenesis through the activation of downstream tumor suppressors and the down-regulation of oncoprotein expression. A study showed that impaired ribosomes induce a p53-dependent cell cycle arrest [42]. RPL5 has also been reported to play tumor suppressor roles in breast tumors [43].

The functions and significance of RPL10 is largely unknown so far. Existing literature mainly focused on its association with autism and is still in debate [44, 45]. There is one report implicating RPL10 in T cell acute lymphoblastic leukemia (T-ALLs). Exome sequencing analysis identified mutation of RPL5 and RPL10 in 12 of 122 (9.8%) pediatric T-ALLs, with a recurrent mutation of Arg98 in RPL10 [46]. Together, these studies point to a potential role of RPL5 and RPL10 in tumorigenesis, although the relevance of both genes in the KRT and AR/RT has not been elucidated.

In this study, we examined the transcriptome profiles to identify novel prognostic markers for RTs, a rare, lethal, mostly pediatric cancer. After identifying differentially expressed lncRNAs and PCGs, we found intense dysregulation in lncRNA-PCG co-expressed pairs in AT/RT and KRT. Among the key cancer-related PCGs in the co-expression network, RPL5 and RPL10 showed high levels of sensitivity and specificity AT/RT and KRT. After comparison with other common pediatric tumors, RPL5 and RPL10 can also be used to distinguish AT/RT from medulloblastoma. To our knowledge, this study is the first in associating RPL5 and RPL10 with AT/RT diagnosis. Our results therefore identify two novel promising diagnostic markers for AT/RT, and provide the basis for work to further assess the performance, and to develop a robust diagnosis practice using these markers.

## Conclusions

Our study mined existing GEO datasets for novel diagnostic markers associated with Rhabdoid tumors, and identified RPL5 and RPL10 as potential diagnostic markers for AT/RT. These two biomarkers may be used as supplementary biomarkers to canonical diagnostic tools such as biopsy and immunohistochemistry. Further research is warranted to characterize the roles and significance of RPL5 and RPL10 in AT/RT.

## Additional files

**Additional file 1: Table S1.** Datasets of the 47 AT/RTs, 31 KRTs, 8 normal brain samples and 23 normal kidney samples.

**Additional file 2: Figure S1.** An overview of PCGs used in this study. There were a total of 12831 PCGs in the microarray profiles (denoted AllPCG), among which 491 differentially expressed PCGs AT/RT vs. Brain, KRT vs. Kidney and KRT vs. AT/RT (denoted DiffPCG). DiffPCGs that have been reported to be strongly associated with cancer (denoted CancerG) were highlighted and provide a pool of candidate diagnostic markers.

**Additional file 3: Figure S2.** Relative expression levels of RPL5 (A, B, and C) and RPL10 (D, E, and F) in AT/RT vs. Brain, KRT vs. Kidney and KRT vs. AT/RT.

## Abbreviations

RT: rhabdoid tumors
AT/RT: Atypical teratoid/rhabdoid tumors
KRT: kidney rhabdoid tumors
PCG: protein coding gene
lncRNA: long non-coding RNA
ROC: receiver operating characteristic
MB: medulloblastoma
CCSK: clear cell sarcoma of the kidney
