# Supplementary figures and images for: Identification of RPL5 and RPL10 as novel diagnostic biomarkers of Atypical teratoid/rhabdoid tumors
Source: Cancer Cell Int. 2018 Nov 20;18:190. doi: 10.1186/s12935-018-0681-1 (PMC6245545; doi:10.1186/s12935-018-0681-1)

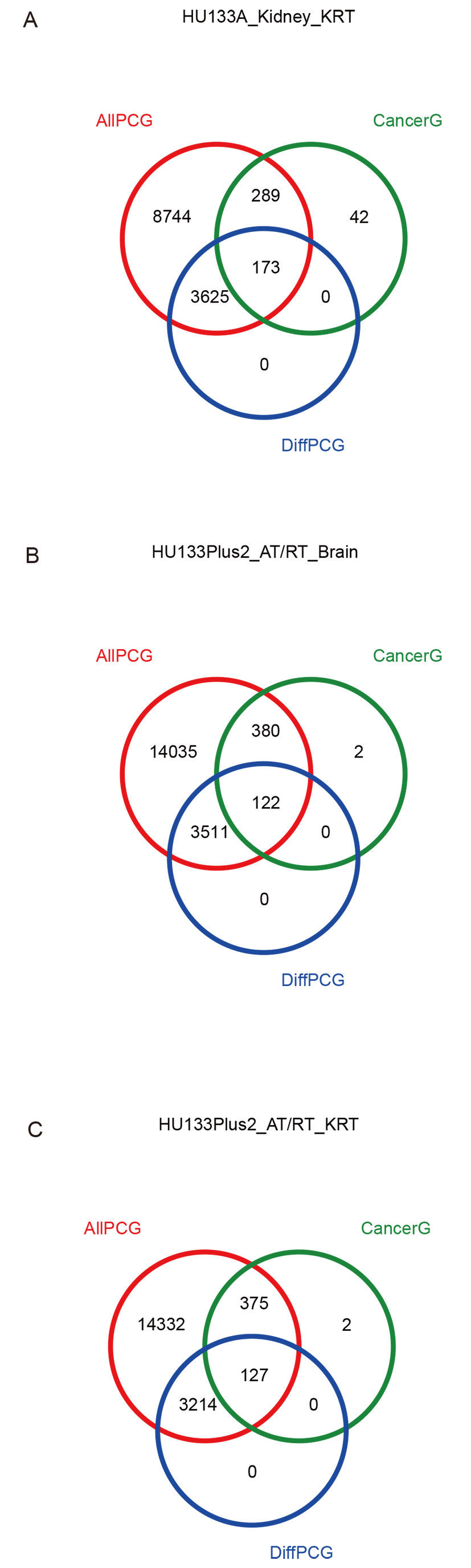

Supplement: Supplementary file 2 — Additional file 2: Figure S1. An overview of PCGs used in this study. There were a total of 12831 PCGs in the microarray profiles (denoted AllPCG), among which 491 differentially expressed PCGs AT/RT vs. Brain, KRT vs. Kidney and KRT vs. AT/RT (denoted DiffPCG). DiffPCGs that have been reported to be strongly associated with cancer (denoted CancerG) were highlighted and provide a pool of candidate diagnostic markers. [file 12935_2018_681_MOESM2_ESM.tif]

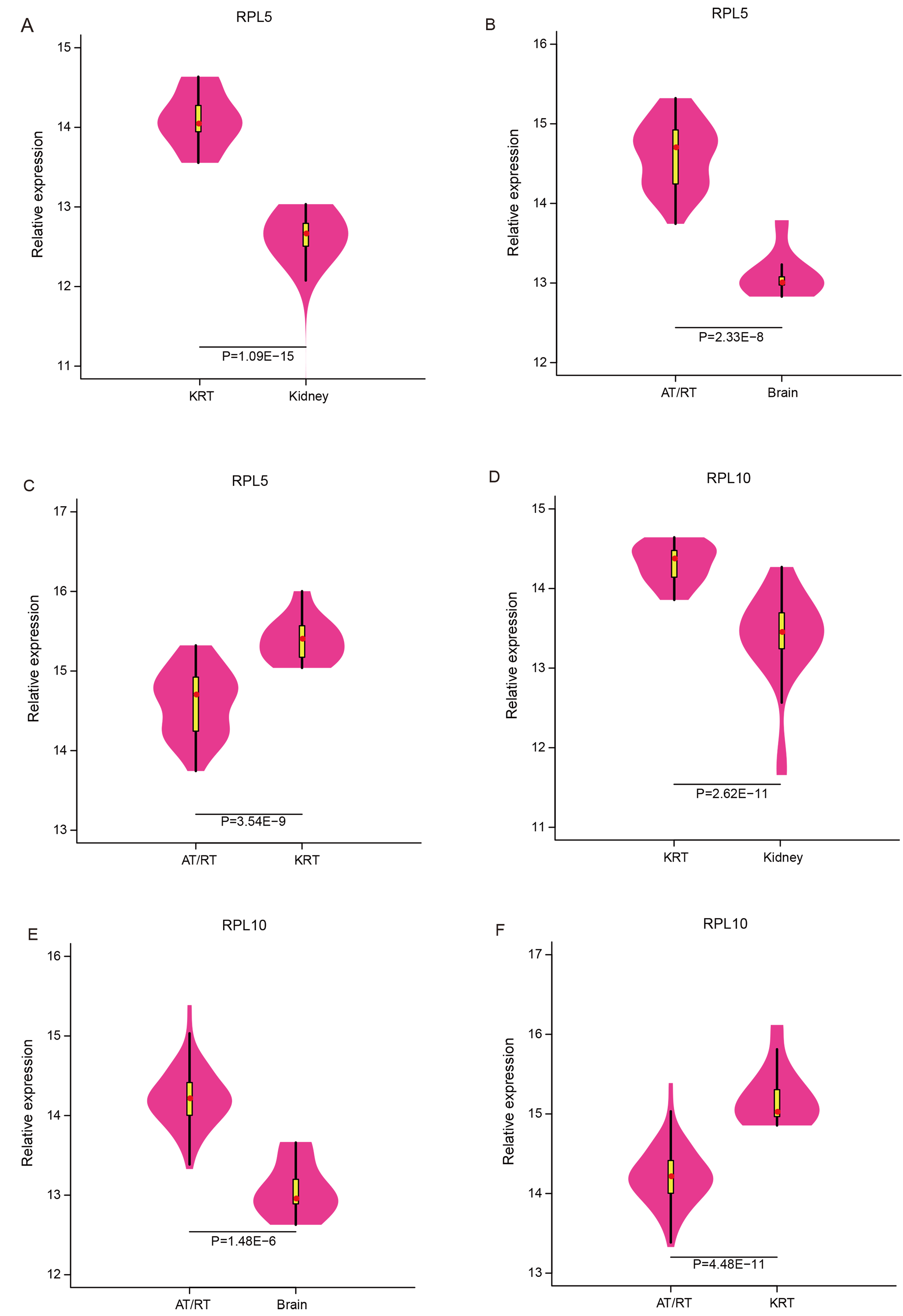

Supplement: Supplementary file 3 — Additional file 3: Figure S2. Relative expression levels of RPL5 (A, B, and C) and RPL10 (D, E, and F) in AT/RT vs. Brain, KRT vs. Kidney and KRT vs. AT/RT. [file 12935_2018_681_MOESM3_ESM.tif]
